# Spatial-temporal heterogeneity of hand, foot and mouth disease and impact of meteorological factors in arid/ semi-arid regions: a case study in Ningxia, China

**DOI:** 10.1186/s12889-019-7758-1

**Published:** 2019-11-08

**Authors:** Jie Li, Xiangxue Zhang, Li Wang, Chengdong Xu, Gexin Xiao, Ran Wang, Fang Zheng, Fang Wang

**Affiliations:** 1 Department of Resources and Environment, Ningxia University, Yinchuan, 750021 China; 2 Ningxia (China-Arab) Key Laboratory of Resource Assessment and Environmental Regulation in Arid Region, Ningxia University, Yinchuan, 750021 China; 3 Faculty of Geographical Science, Beijing Normal University, Beijing, 100875 China; 4 State Key Laboratory of Resources and Environmental Information System, Institute of Geographic Sciences and Natural Resources Research, Chinese Academy of Sciences, 11A, Datun Road, Beijing, 100101 China; 5 College of Environment and Planning, Henan University, KaiFeng, 475001 China; 6 Key Laboratory of Geospatial Technology for the Middle and Lower Yellow River Regions (Henan University), Ministry of Education, Kai Feng, 475001 China; 7 China National Center for Food Safety Risk Assessment, Beijing, 100022 China

**Keywords:** Hand, Foot and mouth disease, Spatial-temporal variation, GeoDetector; Bayesian space-time hierarchical model, Ningxia

## Abstract

**Background:**

The incidence of hand, foot and mouth disease (HFMD) varies over space and time and this variability is related to climate and social-economic factors. Majority of studies on HFMD were carried out in humid regions while few have focused on the disease in arid/semi-arid regions, more research in such climates would potentially make the mechanism of HFMD transmission clearer under different climate conditions.

**Methods:**

In this paper, we explore spatial-temporal distribution of HFMD in Ningxia province, which has an arid/semi-arid climate in northwest China. We first employed a Bayesian space-time hierarchy model (BSTHM) to assess the spatial-temporal heterogeneity of the HFMD cases and its relationship with meteorological factors in Ningxia from 2009 to 2013, then used a novel spatial statistical software package GeoDetector to test the spatial-temporal heterogeneity of HFMD risk.

**Results:**

The results showed that the spatial relative risks in northern part of Ningxia were higher than those in the south. The highest temporal risk of HFMD incidence was in fall season, with a secondary peak in spring. Meteorological factors, such as average temperature, relative humidity, and wind speed played significant roles in the spatial-temporal distribution of HFMD risk.

**Conclusions:**

The study provide valuable information on HFMD distribution in arid/semi-arid areas in northwest China and facilitate understanding of the concentration of HFMD.

## Background

Hand, foot and mouth disease (HFMD) is a common infectious disease, affecting mainly children under 5 years of age, which is caused by a group of enteroviruses, in particular Coxsackievirus A16 and Enterovirus 71. Many widespread epidemics have been reported worldwide since the early 1970s, while the largest outbreaks of HFMD happened in the Asia-Pacific region, for reasons not completely clear [1–3]. HFMD endemic has been reported in Japan since 1967 [4]. In Singapore, nationwide epidemics occurred periodically with the annual incidence rate per 100,000 population increasing from 125.5 in 2001 to 435.9 in 2007 [5]. In China, HFMD prevalence has been reported from 2007, when several large outbreaks of HFMD had occurred [6]. Since 2009, over one million cases of HFMD occurred on a yearly basis and keeps being one of the most infected diseases in China. Environmental risk factors such as climatological factors, population factors and economic condition may affect the incidence of HFMD [7].

The occurrence of HFMD showed apparent seasonality in countries or regions with distinct climate conditions. In country level, previous studies demonstrated that HFMD incidence showed apparent seasonal variation across China, while HFMD cases were prevalent throughout the year. In Northern Thailand, HFMD occurred throughout the year but peaked in the rainy and cold seasons [8]. In regional level, peaks of HFMD were observed in spring and summer (March to July) in Henan province (located in eastern central China) [9]. In Yixing city (one of the biggest cities in southern China in Jiangsu province) HFMD were also found to be most prevalent in summer [10].

Most of the previous studies concentrated in areas with high HFMD incidence, which are often characterized by high population density [11]. Climate in high incidence areas are often temperate to humid, such as Hunan, Guangdong, Guangxi and Henan provinces [12–20]. On the other hand, HFMD in arid/semi-arid areas were seldomly studied. To date (May 2019), only one relevant paper was retrieved by searching “hfmd arid semi-arid” on Pubmed. Similar to other studies, the retrieved paper found that weather factor such as average temperature and relative humidity have significant effect on HFMD [21]. In general, the study on HFMD in arid and semi-arid regions is still limited, more research in such climates would potentially make the mechanism of HFMD transmission clearer under different climate conditions.

Moreover, many previous studies have considered the effect of meteorological factors on HFMD in either temporal or spatial domain in isolation [11, 22]. However, the spatial and temporal effects are often combined in determining the incidence of HFMD. Understanding of spatial-temporal heterogeneity and the combined effects that influence HFMD occurrence would be beneficial for controlling its incidence and spread.

In this study, we analyze the spatial-temporal heterogeneity of HFMD and its relationship with meteorological factors in Ningxia province, which is located in northwest China having a semi-arid climate to shed more light on the mechanisms of HFMD transmission in semi-arid regions. The objectives of this study are: (1) to map county-level spatial-temporal heterogeneity and variation of HFMD risks in Ningxia; (2) to detect areas with high incidences of HFMD (hot spots) and areas with low incidences (cold spots) and quantify their changes in spatial and temporal dimension; and (3) to explore the association of meteorological factors with HFMD incidence in semi-arid areas.

## Methods

### Study region

**Fig. 1:**
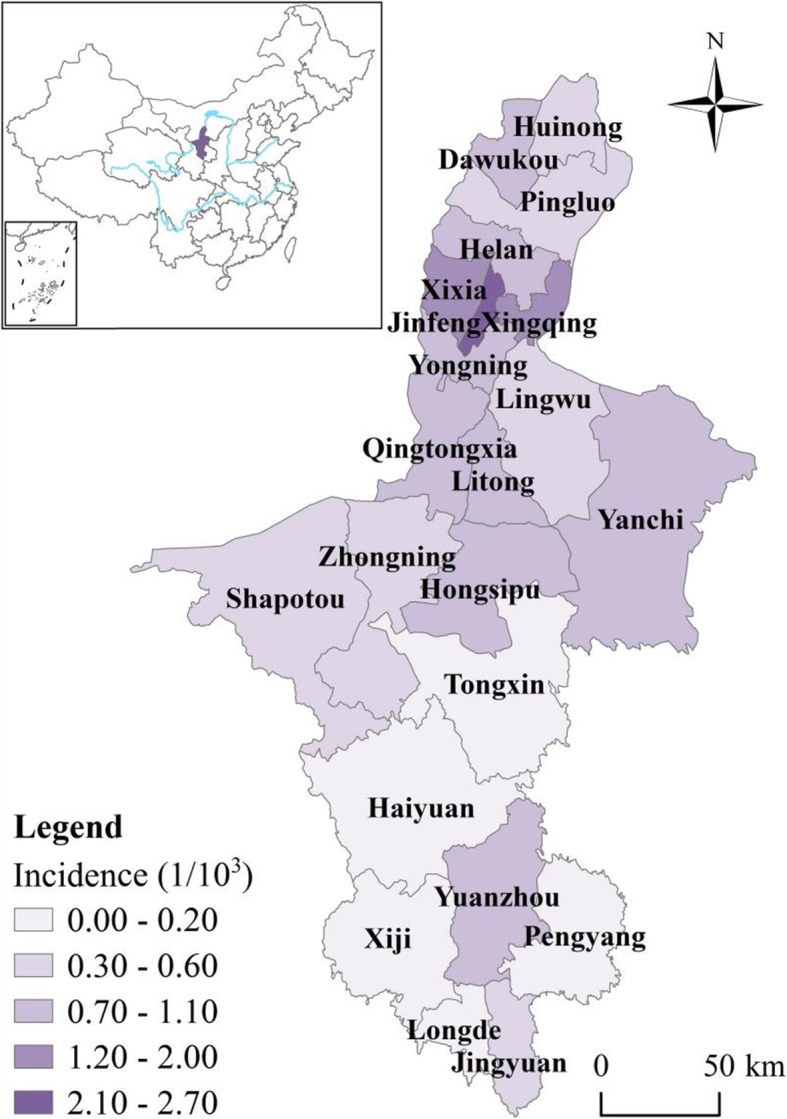
Location of Ningxia province in China and its monthly HFMD incidence (The administrative map in the figure was obtained from the Resource and Environment Data Cloud Platform (http://www.resdc.cn))

Ningxia Hui Autonomous Region (henceforth Ningxia) is located in northwest China (Fig. 1). It has an area of 66,400 km^2^ and total population of about 6.82 million in 2017. The province is mountainous in the south and gradually transforms to flat plain terrain in the north, which has elevation of slight over 1000 m. Ningxia has a typical continental semi-arid climate with annual rainfall around 300 mm and evaporation over 1000 mm. The rainy season is from June to September. Winter in Ningxia is long and cold, while summer is short and hot, with average annual temperature ranges from 5 to 9 °C. The areas with flat terrain in the north are more economically developed and industrialized, while in the southern mountainous areas most counties are underdeveloped, some of which are even listed as state poverty counties of China.

### Data

**Fig. 2:**
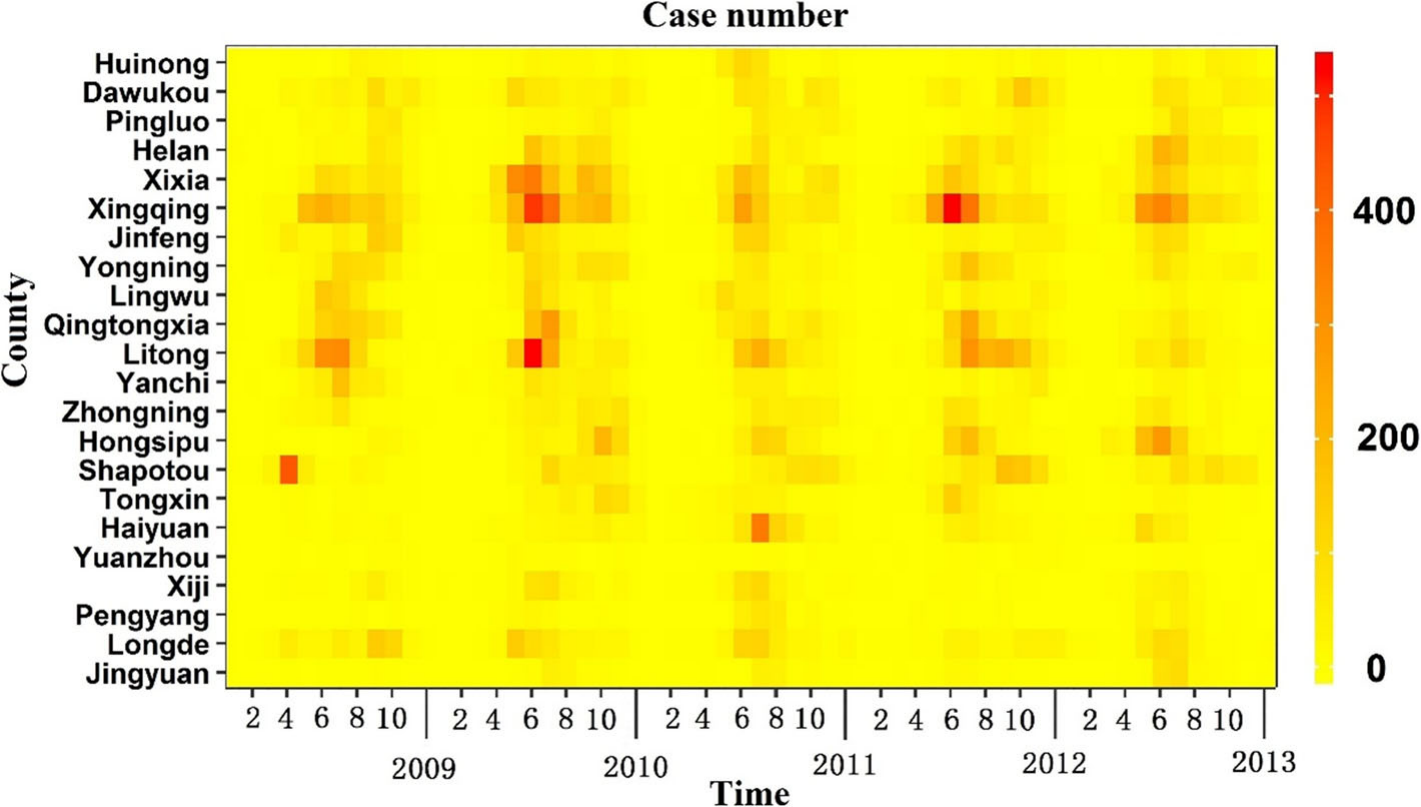
The number of HFMD cases in each county from 2009 to 2013

Data on HFMD were provided by the Chinese Centre for Disease Control and Prevention (CDC). As HFMD is listed as a category C infectious disease, HFMD incidence in China was reported to the CDC via an internet-based reporting system, the national disease report system, by the physicians treating the disease and public health personnel. The data for this study included the number of people diagnosed of HFMD in each county during 1st January 2009 to 31 December 2013 on a monthly basis in Ningxia (Fig. 2).

**Fig. 3:**
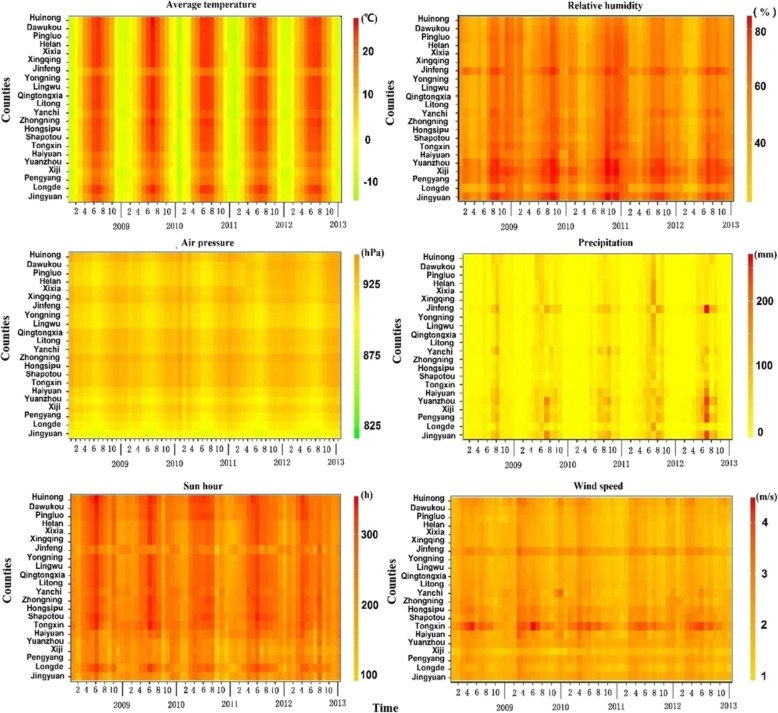
The temporal evolution of meteorological factors from 2009 to 2013

Monthly meteorological data in the same period were obtained from China’s Meteorological Data Sharing Service System (http://data.cma.gov.cn/), which included average temperature, precipitation, relative humidity, air pressure, wind speed, and hours of sunshine based on surveillance stations within Ningxia (Fig. 3).

### GeoDetector

In this study, the spatial and temporal heterogeneity of HFMD risk was calculated by GeoDetector *q* statistic [23–25], as following:

1 $$ q=1-\frac{1}{N{\sigma}^2}{\sum}_{\mathrm{h}=1}^L{N}_h{\sigma}_h^2, $$

where *q* indicates the level of spatial stratified heterogeneity. *q* ranges from zero to one, with zero indicates random distribution and one indicates strong heterogeneity between strata.

### Bayesian space-time hierarchy model

In Bayesian space-time hierarchy model (BSTHM), the number of cases *y*_*it*_ was modeled by the Poisson and log link regression functions, *n*_*it*_ was the risk population [26, 27], following as:

2 $$ {\displaystyle \begin{array}{c}{y}_{it}\sim Poisson\left({n}_{it}{u}_{it}\right)\\ {}\log \left({r}_{it}\right)=a+{s}_i+\left({b}_0{t}^{\ast }+{v}_t\right)+{b}_{1i}{t}^{\ast }+{\sum}_{n=1}^N{\beta}^n{x}_{nit}+{\varepsilon}_{it},\end{array}} $$

where *r*_*it*_ represents the relative risk (RR) of HFMD in region *i* (*i* = 1, 2, …, 22) and month *t* (*t* = 1, 2, …, 60). *α* was the fixed effect. The spatial term *s*_*i*_, represented the disease risk at county *i*. Overall temporal trend was described by (*b*_*0*_t^*^ *+ v*_*t*_), *v*_*t*_ ~ *N* (0, ${\sigma}_v^2$) [28], and *t* ^*^ *t*^∗^=*t* − *t*_*mid*_ indicates the temporal span. The term *b*_*1i*_, quantifies the deviation from the overall temporal variation *b*_*0*_ [9, 27]. The parameter *ε*_*it*_ was noise term.

All counties were classified according to Richardson [29]. That is, a region which the posterior probability *p* (exp (*s*_*i*_) > 1 | data) > 0.80, was defined as a hotspot; whereas less than 0.20, was regarded as a cold spot. The other regions were regarded as neither hot nor cold spots. Then, the regions were further divided depending on the posterior probability *p* (*b*_*1i*_ > 0 |*h*_*i*_, data) of local slope *b*_*1i*_ [27] *b*_*li*_. If a region that the value was greater than 0.8, a faster increasing trend compared with the overall trend, was assumed. If less than 0.2, a slower increasing trend was presented, the remaining are considered to be consistent with the overall trend, which all processes were implemented in the WinBUGS software [30].

## Results

### Spatial-temporal analysis

Between 1st January 2009 to 31st December 2013, 28,446 HFMD cases were reported in Ningxia Province in total. The largest number of cases was recorded in 2010, with an annual incidence of 17.93 per 1000 people. The smallest number of cases was recorded in 2009, with an annual incidence of 11.34 per 1000 people.

**Fig. 4:**
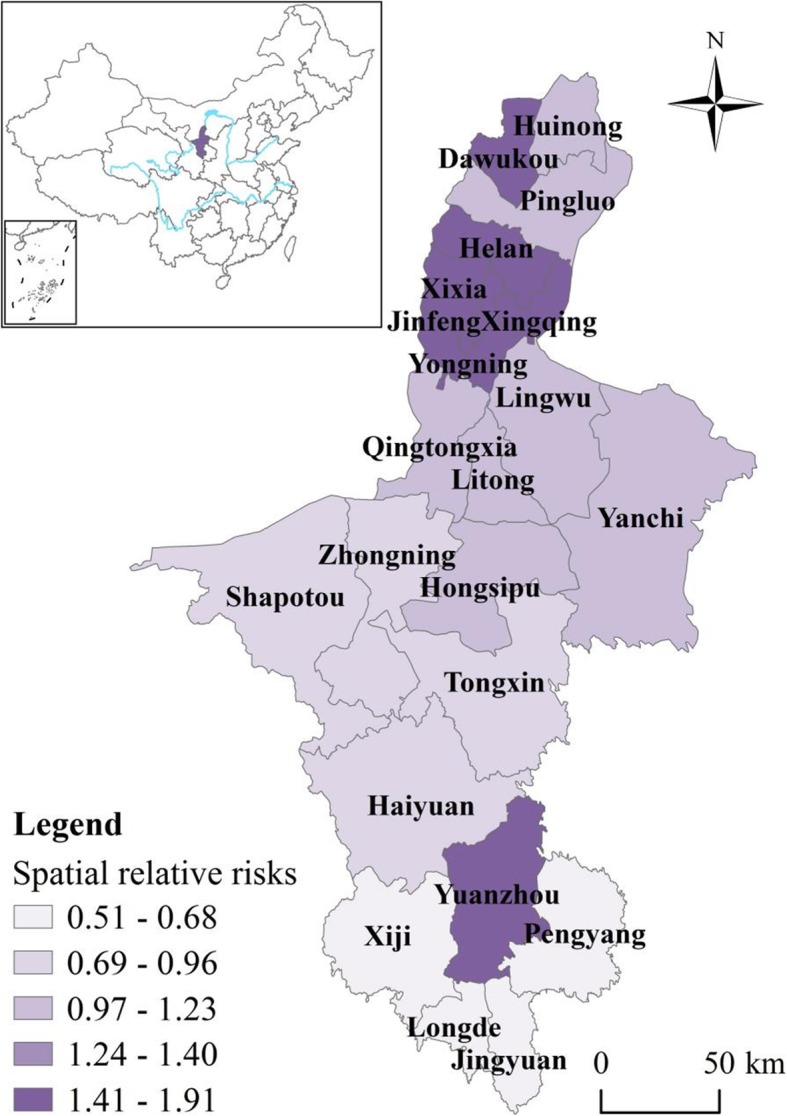
Spatial relative risks (RRs) of HFMD in counties in Ningxia Province (The administrative map in the figure was obtained from the Resource and Environment Data Cloud Platform (http://www.resdc.cn))

There is obvious spatial heterogeneity of the spatial distribution of HFMD in Ningxia as demonstrated by the *q* value of 0.84 calculated by the GeoDetector. Specifically, the spatial RRs in counties in flat northern part of Ningxia were higher (> 1) than the counties in the southern Ningxia (Fig. 4), implying that these counties have relatively higher HFMD risk. Conversely, counties in southern Ningxia generally have lower RRs, except Yuanzhou district which has the highest level of stable spatial RR (> 1.5).

Additionally, the QQ plot was introduced to check the goodness of fit of the actual data and predicted value calculated by BSTHM. It showed that, in Additional file 1: Figure S1, BSTHM performances well to fit the data.

**Fig. 5:**
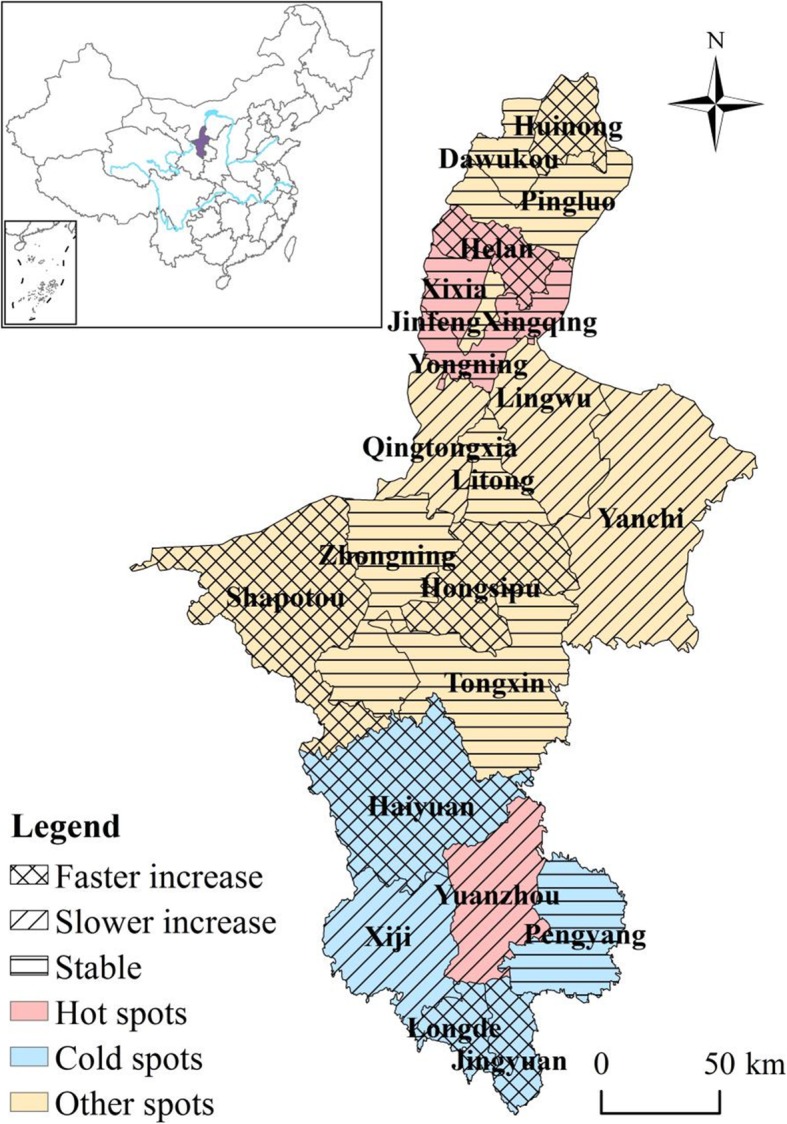
Distribution of hot/cold spots and deviations in local trends compared to the overall trend b1i of HFMD in each county of Ningxia Province (The administrative map in the figure was obtained from the Resource and Environment Data Cloud Platform (http://www.resdc.cn))

As shown in Table 1, five (22.73%) counties out of 22 were classified as hot spots while five counties (22.73%) were classified as cold spots. The rest 12 counties (54.55%) were identified as neither hot nor cold spots. In Fig. 5, the risk of HFMD was relatively higher in northern Ningxia, which is close to Yinchuan, the capital city of Ningxia. Further north to Yinchuan and in the middle of Ningxia the risks were relatively stable. In southern Ningxia, the risk was relatively low except Yuanzhou county, which was a hot spot surrounded by neighboring cold spots. In general, there were hotspot regions distributed both in northern and southern part of Ningxia, irrespective of the economic status.

**Table 1:** Cross-classification of HFMD risk in Ningxia province

	Fast increasing trend	Slow increasing trend	Consistent with global trend	Total
Hot spots	1 (20%)	1 (20%)	3 (60%)	5 (22.73%)
Cold spots	3 (60%)	1 (20%)	1 (20%)	5 (22.73%)
Neither	3 (25%)	3 (25%)	6 (50%)	12 (54.55%)

Helan county further to the north on Yinchuan showed a rapidly increasing trend, while Yuanzhou county located in southern Ningxia showed a slowly increasing trend. Three of the five cold spot counties (Longde, Jingyuan, Haiyuan) exhibited a faster increasing trend while Yuanzhou county showed a slowly increasing trend. Counties that were neither hot nor cold spots, i.e., Huinong, Shapotou and Hongsipu, showed a faster increasing trend while Qingtongxia, Lingwu and Yanchi showed a slower increasing trend (Fig. 5).

**Fig. 6:**
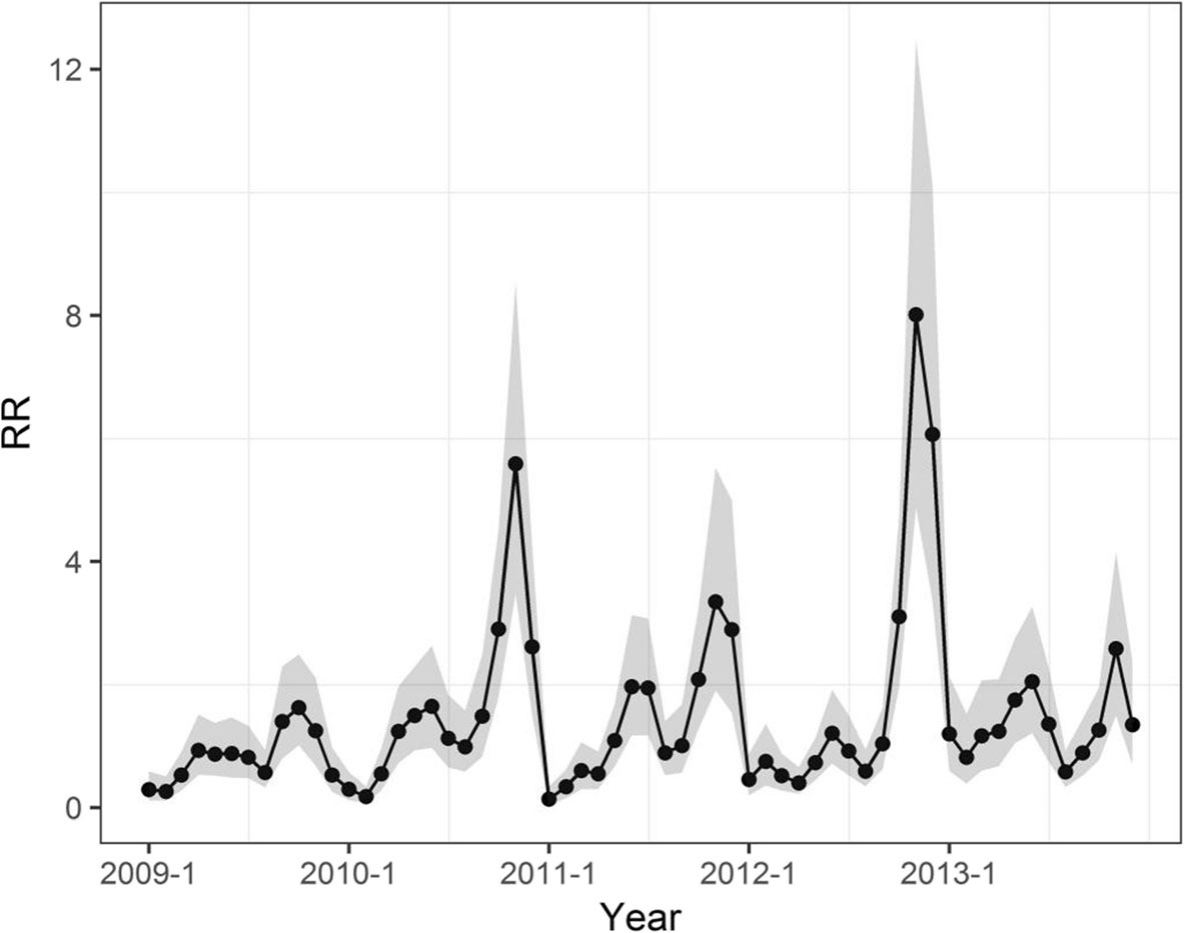
Overall temporal trend of HFMD in Ningxia province (with 95% confidence intervals)

The overall temporal trend of HFMD risk from 2009 to 2013 was non-homogeneous (Fig. 6), the q value was 0.55 according to GeoDetector. The temporal trend in 2009 was relatively low, with dramatic increases in 2010 and 2012. For each year, the highest disease risk occurred in fall season, while the lowest disease risk occurred in winter season. There was also a noticeable secondary peak in summer.

### Meteorological factors analysis

The study reveals that temperature, humidity and wind speed were three dominate factors influencing the transmission of HFMD in the semi-arid regions. There was a positive association between mean temperature and HFMD incidence. An increase of 9.21% (95% CI: 6.65 to 11.67) in the incidence of HFMD occurred with a rise of 1 °C in temperature (RR: 1.10; 95% CI: 1.07 to 1.12). Humidity showed a positive association with HFMD. A 1% increment in relative humidity was associated with a 3.17% increase (95% CI: 1.46 to 4.90) in the incidence of HFMD (RR: 1.03; 95% CI: 1.01 to 1.05). The estimated coefficient for wind speed was not statistically significant, as the parameter’s posterior distribution included zero (Table 2).

**Table 2:** The quantified posterior means and RR of all coefficients

Meteorological factors	Posterior mean (95% CI) (100%)	RR (95% CI)
Mean temperature (°C)	9.21 (6.65–11.67)	1.10 (1.07–1.12)
Relative humidity (%)	3.17 (1.46–4.90)	1.03 (1.01–1.05)
Air pressure (hPa)	1.02 (0.31–1.88)	1.01 (1.00–1.02)
Precipitation (mm)	0.49 (0.19–0.78)	1.005 (1.00–1.01)
Sun hour (h)	0.54 (0.16–0.93)	1.005 (1.00–1.01)
Wind speed (m/s)	−6.91(−37.15–23.4)	0.93 (0.69–1.26)

The other factors also play important roles in the HFMD transmission. Air pressure showed a positive correlation with HFMD. A 1 hPa rise in air pressure were associated with a 1.02% (95% CI: 0.31 to 1.88) increase in the incidence of HFMD (RR: 1.01; 95% CI: 1.00 to 1.02). Precipitation showed a positive correlation with HFMD. A 1 mm increase in precipitation were associated with a 0.49% (95% CI: 0.19 to 0.78) increase in the incidence of HFMD (RR: 1.005; 95% CI: 1.00 to 1.01). Hours of sunshine showed positive relationship with HFMD. A 1 h increase in sunshine hours was associated with rises of 0.54% (95% CI: 0.16 to 0.93) in the incidence of HFMD. The corresponding RRs were 1.005 (95% CI: 1.00 to 1.01) (Table 2).

## Discussion

In this study, a novel BSTHM was used to explore spatial-temporal patterns of HFMD and its association with meteorological factors in Ningxia province, which is semi-arid region in northwest China. Previous studies showed that HFMD mostly concentrate in densely populated areas with humid or temperate climates. However sparely populated areas with arid/semi-arid climate should not be overlooked as it will provide evidence on HFMD distribution in different climate conditions and act as a reference.

In areas with arid/semi-arid climate conditions, the response of HFMD incidence with respect to climate change differs. The results in this paper showed that with a 1 °C increase in temperature, HFMD incidence increased by 9.21%. While in Gansu province with similar climate conditions, a 1 °C increase in temperature resulted in HFMD increase of only 1.8% [21]. This discrepancy indicates that other factors, regardless of climate or socioeconomic, may have played a part in determining the final outcome of HFMD incidence.

The positive correlation between temperature increase and HFMD incidence are corroborated by other studies conducted in areas with different climate conditions. Study conducted in Shanxi province (located in central China) with temperate climate, in Jiangsu province, and Guangzhou city (both are located in south China) also demonstrated similar pattern [13, 31, 32].

However, the positive relation does not hold universally, it is only valid for limited temperature range. For example, a study conducted in Wuhan, the capital city of Hubei province located in south China showed that temperature ranging from 20 to 25 °C prevent HFMD infections in Wuhan [33]. Other climate conditions may also have a threshold effect. A study conducted in mainland China, showed that relative humidity between 80.59 and 82.55% would lead to a higher risk of HFMD [34].

The temporal trends of HFMD in Ningxia varied for different counties. As presented in Table 1, the temporal trend in one (Helan) of five hot spot counties (20%) showed a rapidly increasing trend compared to the overall trend, indicating that Helan county will likely to continue experience a greater RR in the future. As a result, more attention should be paid to prevent the potential HFMD from happening in Helan county. In contrast, one (Yuanzhou) of five hot spot counties (20%) showed a slowly increasing trend in comparison with the overall trend, denoting that HFMD cases will not likely to increase for Yuanzhou district in the future. However, this is not to say that Yuanzhou district is immune from HFMD. On the contrary, because of the large number of HFMD cases in Yuanzhou district, even HFMD cases in Yuanzhou district showed a slowly increasing trend, attention should still be paid to prevent large outbreaks. Finally, the temporal trends in three (Xixia, Xingqing and Yongning) of five hot spot counties (60%) were consistent with the overall trend.

Regarding cold spot counties, three (Longde, Jingyuan, Haiyuan) of five (60%) exhibited a faster increasing trend than the overall increasing trend, indicating that the countries will likely have a high risk of HFMD and even become hot spots in the future. Furthermore, one of the five cold spots counties (20%) showed a slower increasing trend than the overall trend, ndicating that the risk in the county will likely be lower than the overall risk in the future. Finally, the trend in one (Pengyang) of the five cold spot counties (20%) was consistent with the overall trend, indicating that the current risk level in these counties will stay the same in the future.

Of the 12 counties that were neither hot nor cold spots: Huinong, Shapotou and Hongsipu showed a faster local increasing trend than the overall increasing trend, indicating that these counties will likely become hot spots in the future. The public health department should focus its attention on these counties. In addition, three: Qingtongxia, Lingwu and Yanchi, of the 12 counties that were neither hot nor cold spots showed a slower increasing trend compared to the overall increasing trend. Therefore, the counties will probably have a lower RR than the overall risk of other counties in the future, although only by a small margin. Finally, the trend in six of the 12 counties (50%) that were neither hot nor cold spots was consistent with the overall trend.

The relation between climate factors and HFMD incidence is not a straight forward liner relationship, there exists many confounding variables that may potentially influence HFMD incidence. For example, in Ningxia, the spatial distribution of HFMD risk was non-homogeneous, areas with the highest incidence (hot spots) of the disease were mainly concentrated in the northern regions with more developed economy and are more densely populated. Developed economy and favorable living conditions drew large number of internal migrants from underdeveloped southern mountainous areas and their neighboring provinces, where the living conditions are less favorable and economy under-developed. These socioeconomic conditions contributed to the high risk of HFMD in northern part of Ningxia.

Relatively large number of population and more developed economic conditions also contributed to higher risk of HFMD in Yuanzhou county, which stood out as the most disease-prone region in southern Ningxia surrounded by neighboring low risk areas. Similar to Yinchuan, the capital city of Ningxia, Yuanzhou is the economic center in southern mountainous regions and has more favorable living conditions than the neighboring mountainous counties. Furthermore, it has been chosen as the destination of the ecological migrants for the surrounding mountainous areas. Similar studies also found that economically developed and densely populated areas generally have higher risk of HFMD. Wang et al. explored spatiotemporal cluster patterns of HFMD at the county level in mainland China and found that clustering of HFMD were mostly present in eastern and southern China where economy is more developed and population density is high [35]. HFMD rate in urban areas were also found to be higher than in rural areas, corroborating the previous finding [36].

This fact signifies the importance of socioeconomic factors on the incidence of HFMD compared to climate factors. For example, in response to the national strategy of targeted poverty reduction and relocating the poor, large amount of people in underdeveloped mountainous regions have been migrated to areas where the economic and living conditions are better. Yuanzhou district is one of the largest allocation destinations of the ecological migration in mountainous areas. As a result, the incidence of HFMD in Yuanzhou had increased dramatically. This finding was consistent with that of previous studies that population density [11], child density [7], and changes in the size of the population at risk (children 0–14 years old) have influences on HFMD incidence, together with other risk factor such as GDP [37, 38]. However, the influences of these socioeconomic factors are sometimes difficult to quantify than climate factors. Previous study also demonstrated that the performance of health systems is also an important influencing factor of HFMD transmission. When the Health System Performance was low, HFMD transmission increased with the population density, when the Health System Performance was high, HFMD transmission was suppressed [11].

From 2009 to 2013 the incidence of HFMD in Ningxia was generally increasing with varying rate for different counties. However, the rate of change (stable, slower increase, faster increase) did not show a clustering pattern. They showed a scattered pattern across the entire region of Ningxia instead. Furthermore, the rate of change did not correspond with hot/cold spots. In other words, fast increasing trend did not correspond to hot spots while slow increasing trend did not correspond to cold spots. This finding indicated that incidence of HFMD in Ningxia was subject to spatial-temporal heterogeneity, which should be taken into account for the public health department when controlling HFMD in Ningxia.

It should also be noted that although the southern part of Ningxia generally belongs to cold spot regions, the relative risk of HFMD occurrence is increasing. Moreover, the trend of increasing also happened in the middle and northern part of Ningxia, signifying the importance of controlling the HFMD not only in currently high risk areas but also in currently low risk areas to prevent unexpected large outbreaks in the future.

In addition to being spatially non-homogeneous, the risk of HFMD exhibited apparent seasonal variation. The seasonality of HFMD risk also indicated that climate factors likely played an important role in the temporal variance of this disease, as many previous studies indicated [39–41] because temperature may influence the recombination of the pathogenic viruses and the survival of the viruses [42]. In addition, temperatures may be associated with specific human behavioral patterns such as time spend going outside or school time, which could affect the transmission of HFMD [43]. During the entire study period, the highest incidence occurred in fall (September, October and November) and the lowest incidence occurred in winter in Ningxia, which is different from various other studies [4, 8, 13], indicating the influence of climate on HFMD incidence exist difference in different geographic areas. The increased HFMD in fall in Ningxia may be contributed to the climate conditions. As the temperature rises in spring, the risk of HFMD was increasing and reached the secondary peak, but as the temperature continued to rise, the HFMD risk dropped, similar to the threshold effect reported in Wuhan [33]. However, the exact conditions (range of temperature and humidity) that trigger this threshold effect are probably different as Wuhan is located in south China with humid climate.

Moreover, similar studies also found that there was also a secondary peak of HFMD occurrence around spring (April, May, and June). For example, in one of the largest population-based study to date of the epidemiology of HFMD, Xing et al. reported that in China, HFMD peaked annually in June in the North, whereas Southern China experienced semi-annual outbreaks in May, September, and October [44]. Apart from climate factors, the secondary peak may also relate to the concentration of internal migrant workers. After the Chinese New Year, large number of internal migrant workers from rural areas begin to move to more economically developed cities in eastern and southern China in order to make more profit. Large number of migrants potentially increases the risk of HFMD occurrence. In Ningxia province, large amount of migrant workers moves from southern mountainous areas to northern areas in Ningxia and other provinces in China, which is organized by the local government of Ningxia as a remedy for alleviating the poor from underdeveloped mountainous regions. From 2017 to 2020, there is an expected amount of 120,000 such migrants. High concentration of migrant population during spring potentially aggravated HFMD transmission and caused the secondary peak.

Although climate, socioeconomic factors and policy were identified as potential risk factors for the HFMD incidence in Ningxia, the relative importance for each factor and their interaction were not performed, in part due to the difficulty in quantify the influence of policy, health systems and other similar socioeconomic factors. Future studies aiming to explore the influence on the HFMD influence could compare the number of HFMD incidence before and after the policy on promoting local workers working outside Ningxia was implemented. Although it appeared from the result that HFMD in Ningxia is more sensitive to temperature variation compared with other semi-arid, humid or temperate areas, we should be cautious to draw the conclusion as there are other possible confounding variables not controlled. Future studies should therefore try to identify the influence of different risk factors that can potentially influence the HFMD incidence, and to quantify the interactions between the risk factors.

## Conclusions

The findings of our study indicated that the spatial-temporal distribution of HFMD risk in Ningxia Province in China was non-homogeneous. Climate risk factors such as temperature, humidity, and air pressure showed significant correlations with HFMD incidence. Climate and specific human behavioral patterns may have contributed to the peaks of HFMD incidence, but further study is still needed to determine the scale of the effect and exclude other confounding variables.

This study shed more light on the spatial-temporal pattern and variation of HFMD in a representative region with semi-arid climate. The findings can benefit decision-makers in the public health sector and government sector concerning prevention and control the disease. The discovery of secondary peak of HFMD incidence during winter season could further benefit the management of the HFMD in Ningxia and areas with similar physical and socioeconomic characteristics.

## Supplementary information

**Additional file 1: Figure S1.** QQ plot of HFMD incidence for the 22 counties in Ningxia and for the time period from 2009 to 2013. In each plot, points located on the reference red line indicate a perfect agreement with data and function.

## Data Availability

The datasets used and/or analyzed during the current study are available from the corresponding author on reasonable request.

## Abbreviations

BSTHM: Bayesian Space-Time Hierarchy Model
HFMD: Hand, foot and mouth disease
RR: Relative risk
