# Supplementary material for: Spatial-temporal heterogeneity of hand, foot and mouth disease and impact of meteorological factors in arid/ semi-arid regions: a case study in Ningxia, China
Source: BMC Public Health. 2019 Nov 8;19:1482. doi: 10.1186/s12889-019-7758-1 (PMC6839228; doi:10.1186/s12889-019-7758-1)
Supplement: Supplementary file 1 — Additional file 1: Figure S1. QQ plot of HFMD incidence for the 22 counties in Ningxia and for the time period from 2009 to 2013. In each plot, points located on the reference red line indicate a perfect agreement with data and function. [file 12889_2019_7758_MOESM1_ESM.docx]

**Supplementary Material**

**Title:**

Spatial-temporal heterogeneity of hand, foot and mouth disease and impact of meteorological factors in arid/ semi-arid regions: a case study in Ningxia, China

**Author names and affiliations:**

Jie Li^#1,2^, Xiangxue Zhang^#3,4^, Li Wang^5,6^, Chengdong Xu^4,*^, Gexin Xiao^7,*^, Ran Wang^1^, Fang Zheng^1,2^, Fang Wang ^1,2^

^1^Department of Resources and Environment, Ningxia University, Yinchuan, 750021, China

^2^Ningxia (China-Arab) Key Laboratory of Resource Assessment and Environmental Regulation in Arid Region, Ningxia University, Yinchuan, 750021, China

^3^Faculty of Geographical Science, Beijing Normal University, China, 100875

^4^State Key Laboratory of Resources and Environmental Information System, Institute of Geographic Sciences and Natural Resources Research, Chinese Academy of Sciences, Beijing, 100101, China

^5^College of Environment and Planning, Henan University, KaiFeng 475001, China

^6^Key Laboratory of Geospatial Technology for the Middle and Lower Yellow River Regions (Henan University), Ministry of Education, Kai Feng 475001, China

^7^China National Center for Food Safety Risk Assessment, Beijing 100022, China

^*^Corresponding author at: State Key Laboratory of Resources and Environmental Information System, Institute of Geographic Sciences and Natural Resources Research, Chinese Academy of Sciences, 100101, Beijing, China.

Email addresses of corresponding authors: [xucd@lreis.ac.cn](mailto:xucd@lreis.ac.cn); [biocomputer@126.com](mailto:biocomputer@126.com)

^#^Contributed equally.

**Figure**

**Fig. S1** QQ plot of HFMD incidence for the 22 counties in Ningxia and for the time period from 2009 to 2013. In each plot, points located on the reference red line indicate a perfect agreement with data and function.


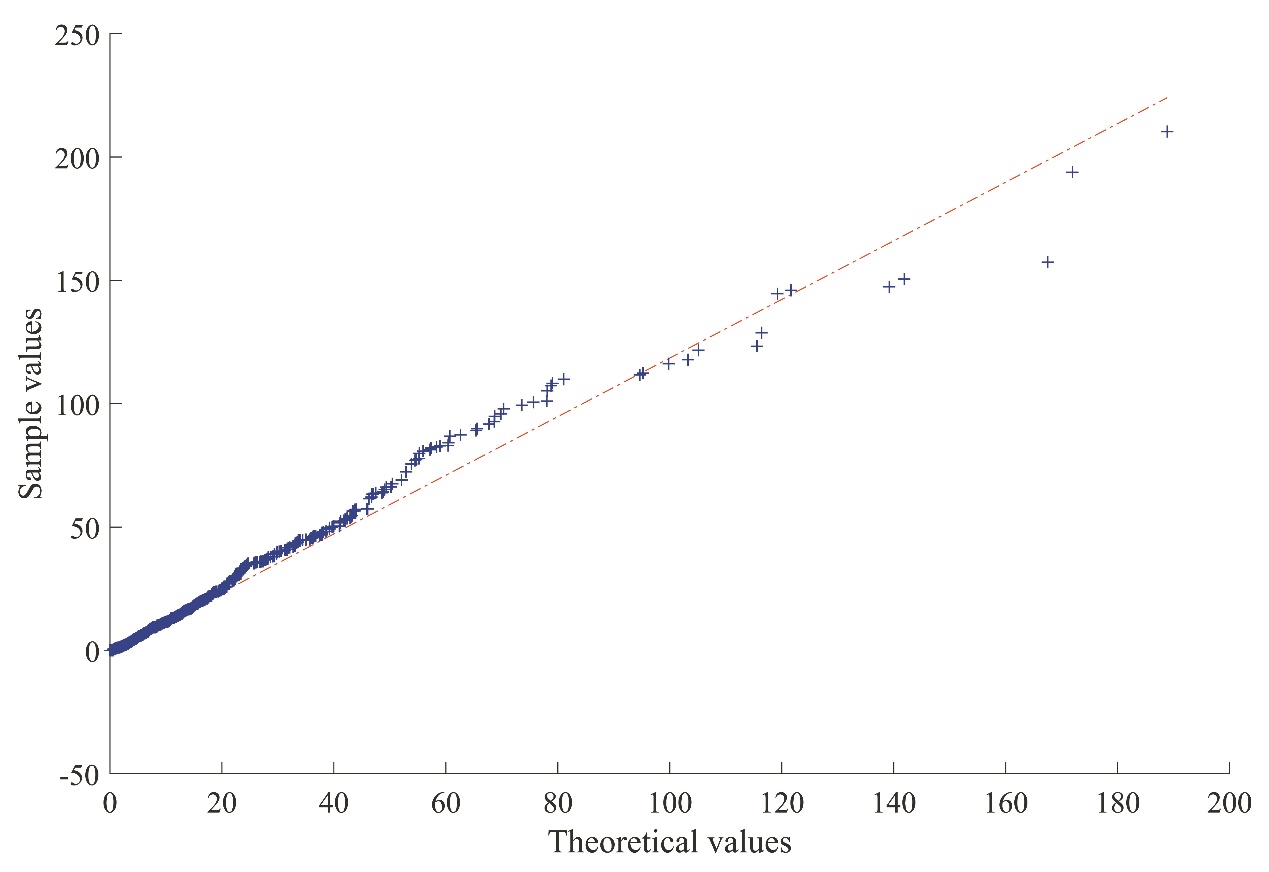


**Fig. S1** QQ plot of HFMD incidence for the 22 counties in Ningxia and for the time period from 2009 to 2013. In each plot, points located on the reference red line indicate a perfect agreement with data and function.
